# Genetic ablation of *Sarm1* attenuates expression and mislocalization of phosphorylated TDP-43 after mouse repetitive traumatic brain injury

**DOI:** 10.1186/s40478-023-01709-4

**Published:** 2023-12-20

**Authors:** Elif O. Dogan, James Bouley, Jianjun Zhong, Ashley L. Harkins, Allison M. Keeler, Daryl A. Bosco, Robert H. Brown, Nils Henninger

**Affiliations:** 1 Department of Neurology, University of Massachusetts Chan Medical School, 55 Lake Ave, North, Worcester, MA 01655 USA; 2 Department of Neurosurgery, The First Affiliated Hospital of Chongqing Medical University, Chongqing, China; 3 Graduate Program in Neuroscience, Morningside Graduate School of Biomedical Sciences, University of Massachusetts Chan Medical School, Worcester, MA 01655 USA; 4 Horae Gene Therapy Center, University of Massachusetts Chan Medical School, Worcester, MA 01605 USA; 5 Department of Pediatrics, University of Massachusetts Chan Medical School, Worcester, MA 01605 USA; 6 NeuroNexus Institute, University of Massachusetts Chan Medical School, Worcester, MA 01605 USA; 7 Department of Psychiatry, University of Massachusetts Chan Medical School, 55 Lake Ave, North, Worcester, MA 01655 USA

**Keywords:** Axon, Behavior, Brain injury, Glial scar, Haploinsufficiency, Interleukin, Neurodegeneration, SARM1, Tau, TDP-43

## Abstract

Traumatic brain injury (TBI), particularly when moderate-to-severe and repetitive, is a strong environmental risk factor for several progressive neurodegenerative disorders. Mislocalization and deposition of transactive response DNA binding protein 43 (TDP-43) has been reported in both TBI and TBI-associated neurodegenerative diseases. It has been hypothesized that axonal pathology, an early event after TBI, may promote TDP-43 dysregulation and serve as a trigger for neurodegenerative processes. We sought to determine whether blocking the prodegenerative *Sarm1* (sterile alpha and TIR motif containing 1) axon death pathway attenuates TDP-43 pathology after TBI. We subjected 111 male *Sarm1* wild type, hemizygous, and knockout mice to moderate-to-severe repetitive TBI (rTBI) using a previously established injury paradigm. We conducted serial neurological assessments followed by histological analyses (NeuN, MBP, Iba-1, GFAP, pTDP-43, and AT8) at 1 month after rTBI. Genetic ablation of the *Sarm1* gene attenuated the expression and mislocalization of phosphorylated TDP-43 (pTDP-43) and accumulation of pTau. In addition, *Sarm1* knockout mice had significantly improved cortical neuronal and axonal integrity, functional deficits, and improved overall survival after rTBI. In contrast, removal of one *Sarm1* allele delayed, but did not prevent, neurological deficits and neuroaxonal loss. Nevertheless, *Sarm1* haploinsufficient mice showed significantly less microgliosis, pTDP-43 pathology, and pTau accumulation when compared to wild type mice. These data indicate that the *Sarm1*-mediated prodegenerative pathway contributes to pathogenesis in rTBI including the pathological accumulation of pTDP-43. This suggests that anti-*Sarm1* therapeutics are a viable approach for preserving neurological function after moderate-to-severe rTBI.

**Supplementary Information:**

The online version contains supplementary material available at 10.1186/s40478-023-01709-4.

## Introduction

Traumatic brain injury (**TBI**) is a global health priority, affecting more than 50 million individuals each year and frequently causing lasting disability [12, 53]. Supported by epidemiological evidence, it is now widely accepted that TBI, particularly when repetitive or moderate-to-severe, can initiate or accelerate chronic neurodegeneration beyond the immediate effects of the acute injury and serves as an important risk factor for several progressive neurodegenerative disorders [11, 24, 83]. Pathological evidence linking TBI with these chronic neurodegenerative diseases includes axonal degeneration as well as mislocalization and deposition of transactive response DNA binding protein 43 (**TDP-43**) [17, 49, 83].

Axonal pathology is an early event after TBI [18] that may promote TDP-43 dysregulation [38, 57], possibly serving as a trigger for neurodegenerative processes [17, 24]. It has been shown that activation of the SARM1 (sterile alpha and TIR motif containing 1) protein drives a general axonal destruction program in several categories of neuronal injury including TBI that is alleviated by genetic ablation of *Sarm1* [7, 10, 26, 51, 52], rendering it an attractive therapeutic target. Under physiologic conditions, nicotinamide nucleotide adenylyltransferase 2 (**NMNAT2**) is an axon survival factor that generates nicotinamide adenine dinucleotide (**NAD+**) from nicotinamide mononucleotide (**NMN**). After disruptions to axon transport, NMNAT2 is depleted from the distal axon, NAD + drops, and NMN rises. The rise in NMN/NAD + ratio triggers a conformational change of SARM1 via binding to an allosteric site on SARM1, releasing its autoinhibitory HEAT/Armadillo motifs (**ARM**) domain and activating SARM1. Once activated, SARM1 can drive further NAD + depletion through an intrinsic NAD + hydrolase activity in its Toll-interleukin-1 receptor (**TIR**) domain. Current models propose that SARM1 NADase activity drives axon destruction in injured axons [1, 20, 66].

We previously demonstrated that moderate-to-severe repetitive TBI (**rTBI**) causes pathological mislocalization of both TDP-43 and phosphorylated TDP-43 (**pTDP-43**), neuronal and axonal degeneration, and functional deficits in mice [33]. Interestingly, *Sarm1* knockout has been shown to attenuate TDP-43–linked motor neuron degeneration [85]. In humans, TDP-43 prevents the mis-splicing of the axonal maintenance factor stathmin2 (STMN2) [3, 22, 37, 55, 80], which co-migrates with NMNAT2 in axons, and it is co-regulated with NMNAT2 by MAPK stress signaling [73, 74, 80]. These observations lead to the hypothesis that STMN2 may function upstream of SARM1 [80], providing a possible explanation how after trauma, when TDP-43 is dysfunctional, SARM1 could be disinhibited and activate axonal degradation. Nevertheless, recent observations indicated that *Sarm1* knockout does not rescue the motor phenotype of mice lacking STMN2, indicating that STMN2 does not regulate the activity of SARM1 [39]. Accordingly, it remains to be clarified whether genetic inactivation of *Sarm1* may be a promising strategy to attenuate TBI-associated neurodegeneration associated with TDP-43 pathology.

To gain further insight into this issue, we used a previously established mouse model of moderate-to-severe rTBI [33] to determine the effect of *Sarm1* depletion on TDP-43 pathology. To understand the impact of complete versus partial *Sarm1* inactivation, an important issue from a therapeutic standpoint as pharmacological interventions are unlikely to completely inactivate SARM1, we used both *Sarm1* knockout (*Sarm1*^−/−^) and *Sarm1* haploinsufficient (*Sarm1*^+/−^)  mice. We show that genetically blocking the endogenous SARM1-mediated axon death pathway significantly attenuated expression and mislocalization of pTDP-43 after repetitive TBI. Reduced TDP-43 pathology was accompanied by improved neuronal and axonal integrity, reduced glial scar formation, as well as significantly improved survival and neurological function. Finally, whilst *Sarm1* haploinsufficiency reduced TDP-43 pathology, functional deficits were only delayed and there was no significant improvement in mortality, neuronal, and axonal degeneration.

## Materials and methods

*Sarm1*^+/−^ males and females on the C57BL6/J background (RRID:IMSR_JAX:018069) were bred to obtain age-matched, male *Sarm1*^+/+^, *Sarm1*^+/−^, and *Sarm1*^−/−^ littermate mice [26]. Animals were socially housed in same-sex groups (n = 4 per cage) on 12-h light/dark cycle with food and water *ad libitum* in a specific pathogen free barrier facility.

We previously showed that wild-type C57BL/6 mice subjected to repetitive sham surgery do not exhibit any neurological deficits or cerebral pathology at 1 month after surgery [33]. Here we investigated whether complete and partial blockade of *Sarm1* signaling suppresses neurological defects associated with repetitive moderate-to-severe TBI. We subjected 111 male *Sarm1*^+/+^ (n = 37), *Sarm1*^+/−^ (n = 37), and *Sarm1*^−/−^ (n = 37) littermate mice to rTBI. In addition, 3 mice per group were subjected to sham injury to serve as controls for the behavioral and histological analyses.

### Closed skull moderate-to-severe rTBI

rTBI was produced by closed skull impact onto the unrestrained head to allow for head acceleration post impact using a weight drop device as described [33]. While there is no universally accepted definition of moderate-to-severe TBI in mice, our model causes a range of cerebral pathologies that are seen in human TBI as well as significant long‑term functional deficits that have been considered consistent with a moderate-to-severe injury [33, 71, 79].

For this study, male mice (age 8–12 weeks; 28.9 ± 2.8 g body weight) were anesthetized with isoflurane in room air. Anesthesia was discontinued immediately prior to each impact and sham injury. Body temperature was monitored continuously with a rectal probe and maintained at 37.0 ± 0.5 °C. For analgesia, animals received 1.5 mg/kg subcutaneous buprenorphine (Med-Vet International, Mettawa, Il, USA) 30 min before anesthesia and every 6 h afterwards until 24 h after the last injury. Additionally, each animal received 5 mg/kg subcutaneous carprofen (Patterson Veterinary, Devens, MA, USA) prior to each injury. Following each TBI, the bone was visually inspected under the operating microscope and animals with a skull fracture euthanized and removed from the study. The wound was closed with interrupted sutures and the animal returned to its home cage after recovery from anesthesia.

### Behavioral testing

Presence of seizure activity was evaluated clinically (facial twitching as well as tail, forelimb, and hindlimb tonic-clonic or tonic movements) as previously detailed [26]. The duration of the loss of the righting reflex was defined as the time (s) it took an animal to spontaneously right itself from a supine to prone position after discontinuation of anesthesia. The neurological severity score (**NSS**) was assessed on a scale from 0 (no deficit) to 10 (maximal deficit) prior to rTBI as well as serially until euthanasia with minor modifications from the original protocol as described [26].

### Immunohistochemistry

Animals were perfused under anesthesia through the ascending aorta with 50 mL saline and then with ice cold phosphate-buffered 4% paraformaldehyde for 10 min. Brains were removed, postfixed overnight in the same fixative and then stored in 0.4% paraformaldehyde at 4 °C until further processing. Prior to paraffin embedding brains were pre-sectioned using a brain matrix. For histological assessment paraffin sections, 10-μm thick coronal, were obtained at approximately Bregma − 2.5 mm (impact center), as described [33]. Immunohistochemistry was performed against pTDP-43^Ser − 409/410^ (Proteintech, 1:250, Cat# 22309-1-AP, RRID: AB_11182943), pTau^Ser−202/Thr205^ (AT8, 1:250, Thermo Fisher Scientific, Cat# MN1020, RRID: AB_223647), neuronal nuclei (**NeuN**, 1:200, Proteintech, Cat# 26975-1-AP, RRID: AB_2880708), myelin basic protein (**MBP**, 1:200, Santa Cruz Biotechnology, Cat# M3821, RRID: AB_1841021), glial fibrillary acidic protein (**GFAP**, 1:250, Agilent, Cat# Z0334, RRID: AB_10013382), and ionized calcium binding adaptor molecule 1 (**Iba-1**, 1:250, Wako, Cat# 019-19741, RRID: AB_839504). For chromogenic staining, tissue sections labeled with the primary antibodies (NeuN) were incubated with appropriate biotin-conjugated secondary antibodies followed by avidin-biotin complex (Vector Laboratories) incubation and treatment with diaminobenzidine as directed by the manufacturer. For immunofluorescence staining tissue sections labeled with the primary antibodies (pTDP-43, pTau, NeuN, MBP, GFAP, Iba-1) were incubated in appropriate secondary antibodies conjugated with Alexa Fluor 488 (1:250, Abcam, Cat# ab150113, RRID: AB_2576208 and Cat# ab150077, RRID: AB_2630356), Alexa Fluor 555 (1:250, Abcam, Cat# ab150106, RRID: AB_2857373), and Alexa Fluor 647 (1:250, Abcam, Cat# ab150075, RRID: AB_2752244 and Cat# ab150115, RRID: AB_2687948). Omitting the primary antibody in a subset of slides served as negative controls.

### Luxol fast blue staining

After deparaffinization and hydration steps, coronal sections were immersed in Luxol fast blue (**LFB**) at room temperature overnight. Differentiation steps were performed using lithium carbonate and 70% ethanol. Following dehydration, slides were mounted with resinous medium.

### Image acquisition and quantification

To acquire images of all stained sections for offline analysis, we used a Leica DM6 B microscopy system equipped with a brightfield DMC5400 color CMOS camera and an immunofluorescent DFC9000 sCMOS camera. All histological analyses were performed by an investigator masked to the animal groups (E.O.D.).

For quantitative thresholded area measurements of histological data, we used the Analyze Particle tool in ImageJ as described [33]. To determine the extent of neuronal loss, chromogen stained NeuN-positive cells were assessed in each coronal section. Images of 16 nonoverlapping regions of interest (**ROI**; 8 per hemisphere; 570 × 375 μm, each) covering the dorsal cerebral cortex were taken at 5x magnification. To assess the impact of rTBI on axonal integrity, we used fluorescence staining for MBP to quantify the signal in the cerebral cortex (one ROI per hemisphere; 1050 × 450 μm, each) and corpus callosum (two ROIs per hemisphere; 56,000 μm^2^, each). In addition, we measured the corpus callosum thickness in the mid-sagittal plane using MBP and LFB stained images to quantify the degree of atrophy.

To assess microgliosis and astrocytosis in the dorsal cortex, we quantified the total thresholded area (μm^2^) of the Iba-1 and GFAP stained area in 16 nonoverlapping ROIs (8 per hemisphere; 600 × 380 μm, each) covering the dorsal cerebral cortex (images taken at 10x magnification). For thresholded area measurement of pTDP-43, images of 14 ROIs (7 per hemisphere; 600 × 380 μm, each) centered within the corresponding ROI used for the GFAP and Iba-1 analyses were taken at 10x magnification and analyzed as described for GFAP. The number of cells expressing pTDP-43 and pTau were quantified in two ROIs (one per hemisphere, 333 × 333 μm each) taken at 40x magnification. Cytoplasmatic pTDP-43 mislocalization was determined in 2 ROIs (one per hemisphere, 666 × 666 μm each) taken at 20x magnification using the ImageJ JACoP plugin. Stained regions of confocal images were selected by setting a single common threshold intensity for all images for a particular staining method.

### Cytometric bead array assay

Under deep isoflurane anesthesia whole blood (500–800 μl) was collected from the right ventricle into Eppendorf tubes containing 6 μl ethylenediaminetetracetic acid (**EDTA**). Samples were immediately centrifuged at 3000 g for 15 min at 4 °C and the layer containing plasma immediately removed and stored in low bind Eppendorf tubes at − 80 °C. For the detection of cytokines in plasma, Cytometric Bead Array assay was performed as previously published [41] except using Biolegend LEGENDplex Mouse Macrophage/Microglia Panel (13-plex) with V-bottom Plate (Biolegend, Cat# 740,846). Plasma samples from the animals were diluted 1:2. Data were acquired on a BD LSRII and analyzed using the LEGENDplex Data Analysis Software Suite.

### Statistical analysis

Unless otherwise stated, continuous variables are reported as mean ± standard error of the mean. Normality of data was examined using the Shapiro–Wilk test. Between group comparisons were conducted by one-way analysis of variance (ANOVA) with post-hoc Holm-Šídák test or ANOVA on Ranks with post-hoc Dunn’s test. Between-group comparisons of continuous variables over repeated measurements (time or ROI) were conducted using longitudinal mixed models with *post-hoc* False Discovery Rate (**FDR**) adjustment. Time (or ROI) was treated as a categorical variable. The models included group and time (or ROI) as fixed covariates, as well as the group × time (or ROI) interactions. Correlation analyses were conducted using Spearman’s rho. Survival analysis was conducted by Kaplan Meier analysis and log-rank test with *post-hoc* Bonferroni protection. The distribution of pTDP-43, pTau, and pTDP-43/pTau double stained cells in the experimental groups was compared by χ^2^-square test with *post-hoc* Bonferroni protection. Two-sided significance tests were used and a two-sided *p* < 0.05 was considered statistically significant. All statistical analyses were performed using IBM® SPSS® Statistics Version 26 (IBM®-Armonk, NY).

## Results

### Sham mice had no functional phenotype or developed neuropathology

Consistent with our previously described model characteristics [33], no sham operated mouse had neurological deficits, seizures, died or exhibited neuronal and axonal loss, microgliosis, astrogliosis, evidence of pTau expression, or pTDP-43 mislocalization (not shown).

### Improved functional phenotype in ***Sarm1***^-/-^ mice after rTBI

**Fig. 1:**
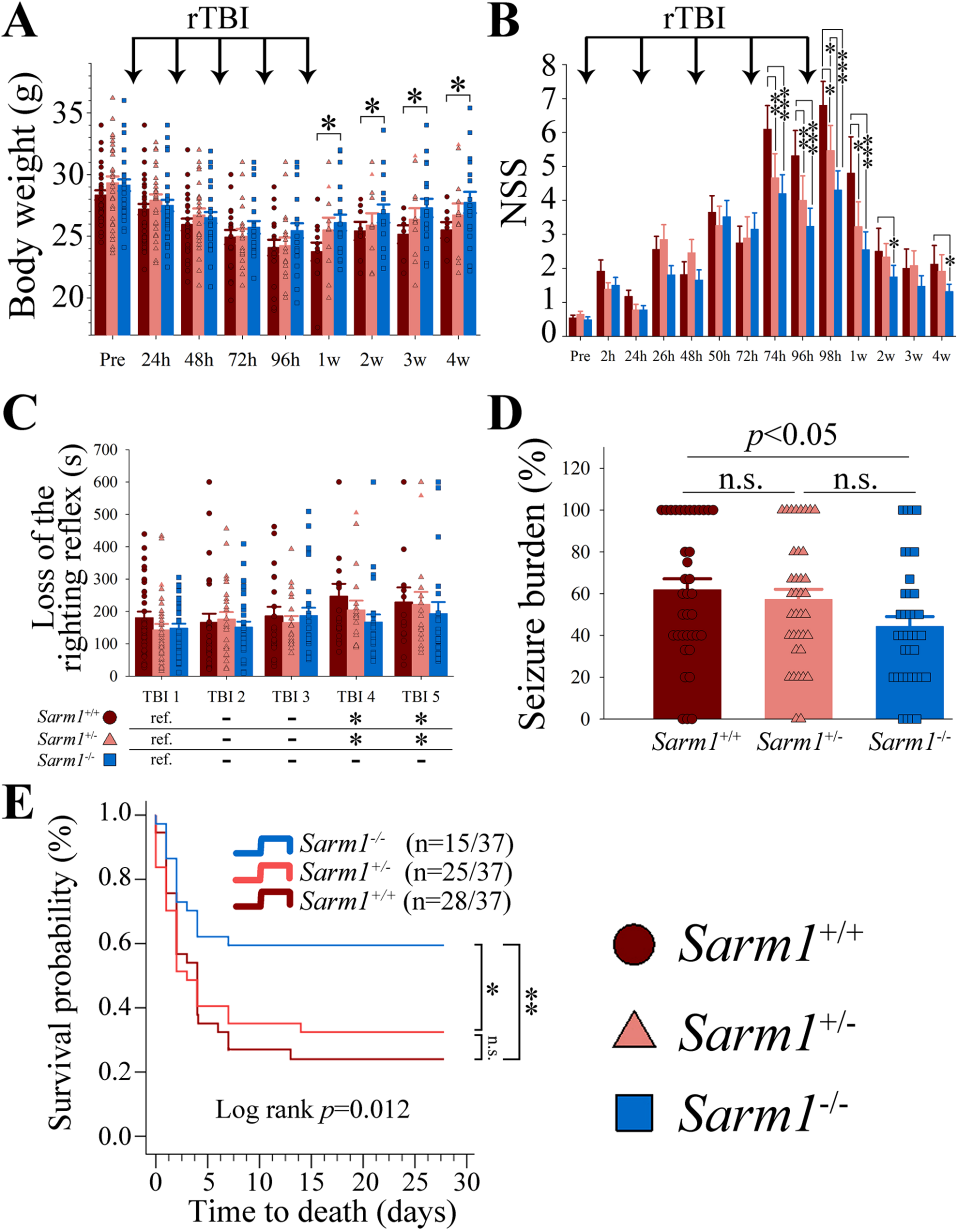
Genetic ablation of *Sarm1*, but not *Sarm1* haploinsufficiency, mitigates rTBI-associated functional deficits and mortality. (**A**) While all rTBI groups lost weight after rTBI, *Sarm1*^−/−^ mice recovered their weight faster than *Sarm1*^*+/+*^ and *Sarm1*^+/−^*mice* (*p* = 0.003 for group effects, *p* < 0.001 for time effects, *p* < 0.001 for group x time interaction). (**B**) While neurological deficits were significantly attenuated in both *Sarm1*^−/−^ and mice *Sarm1*^+/−^ up to 1 week post rTBI, only *Sarm1*^−/−^ mice showed persistent protection up to the 4-week time point (*p* = 0.003 for group effects, *p* < 0.001 for time effects, *p* < 0.001 for group x time interaction). (**C**) Successive rTBI prolonged the time of the return of the righting reflex without difference between groups (group effect *p* = 0.219, time effect *p* = 0.016, group x time *p* = 0.600; *p < 0.05 versus TBI 1 [ref.]). (**D**) *Sarm1*^−/−^ mice had a significantly lower seizure burden when compared to wild type mice (ANOVA on Ranks with *post-hoc* Tukey test). (**E**) Genetic ablation of *Sarm1* significantly reduced rTBI-associated mortality. Numbers in parenthesis indicate the number of mice that died per the total number of mice in each group. Data in bar graphs are mean ± sem. *p < 0.05, **p < 0.01, ***p < 0.001

Similar to our previous observations [34], rTBI mice significantly lost weight after the first injury with a nadir after the last TBI and subsequent partial recovery. However, we found that *Sarm1*^−/−^ mice regained weight more quickly than *Sarm1*^+/+^ mice (*p* < 0.001 for time effects, *p* = 0.106 for group effects, *p* = 0.034 for group x time interaction) (Fig. 1A).

To examine the temporal evolution of functional deficits, we used the NSS, which is a composite of ratings measuring a combination of overall inquisitiveness, postural stability, and motor function. Consistent with our previous observations, there was no change in the NSS over time in sham operated animals (not shown) [26, 33, 34]. In contrast, we observed that in all rTBI groups, mice developed significant neurological deficits after the first impact (2 h time point) that worsened with each subsequent impact injury. After the last TBI (98-hour time point) mice partially improved over time but all groups showed residual neurological deficits up to the 1-month time point when compared to baseline. Importantly, genetic ablation of *Sarm1* significantly attenuated neurological deficit severity when compared to *Sarm1*^*+/+*^ mice (*p* = 0.003 for group effects, *p* < 0.001 for time effects, *p* < 0.001 for group x time interaction). Although *Sarm1* haploinsufficiency also attenuated behavioral dysfunction when compared to *Sarm1*^*+/+*^ mice, this effect was only transient up to the 1-week time point and there was no overall difference compared to *Sarm1*^−/−^ mice (*p* = 0.166) (Fig. 1B).

We observed an increasing duration of loss of the righting reflex with subsequent impacts without difference between groups (*p* = 0.219 for group effects, *p* = 0.016 for time effects, *p* = 0.600 for group x time interaction), indicating that anesthetic effects were unlikely to have contributed to the observed between-group differences in the NSS (Fig. 1C).

Interestingly, we found that most rTBI mice developed impact seizures in response to the delivered impact (*Sarm1*^+/+^ 91.9%, *Sarm1*^+/−^ 94.6%, *Sarm1*^−/−^ 89.2%) without significant between-group difference. However, after accounting for premature death, we found that *Sarm1*^−/−^ mice had a significantly lower seizure burden (seizure burden = number of seizures / number of impacts * 100) than *Sarm1*^+/+^ mice (*p* < 0.05) (Fig. 1D).

Strikingly, we found that genetic ablation of *Sarm1* significantly reduced rTBI-associated mortality (Log Rank *p* = 0.012). On pairwise testing, *Sarm1*^−/−^ mice had a significantly greater survival when compared to both *Sarm1*^+/+^ (*p* = 0.004) and *Sarm1*^+/−^ (*p* = 0.016) mice (Bonferroni adjusted). Though numerically fewer *Sarm1*^+/−^ mice died as compared to *Sarm1*^+/+^ mice, this effect was not statistically significant (*p* = 0.746) (Fig. 1E).

### ***Sarm1*** knockout, but not ***Sarm1*** haploinsuficiency, mitigates cortical neuronal and axonal loss at 1 month after rTBI

**Fig. 2:**
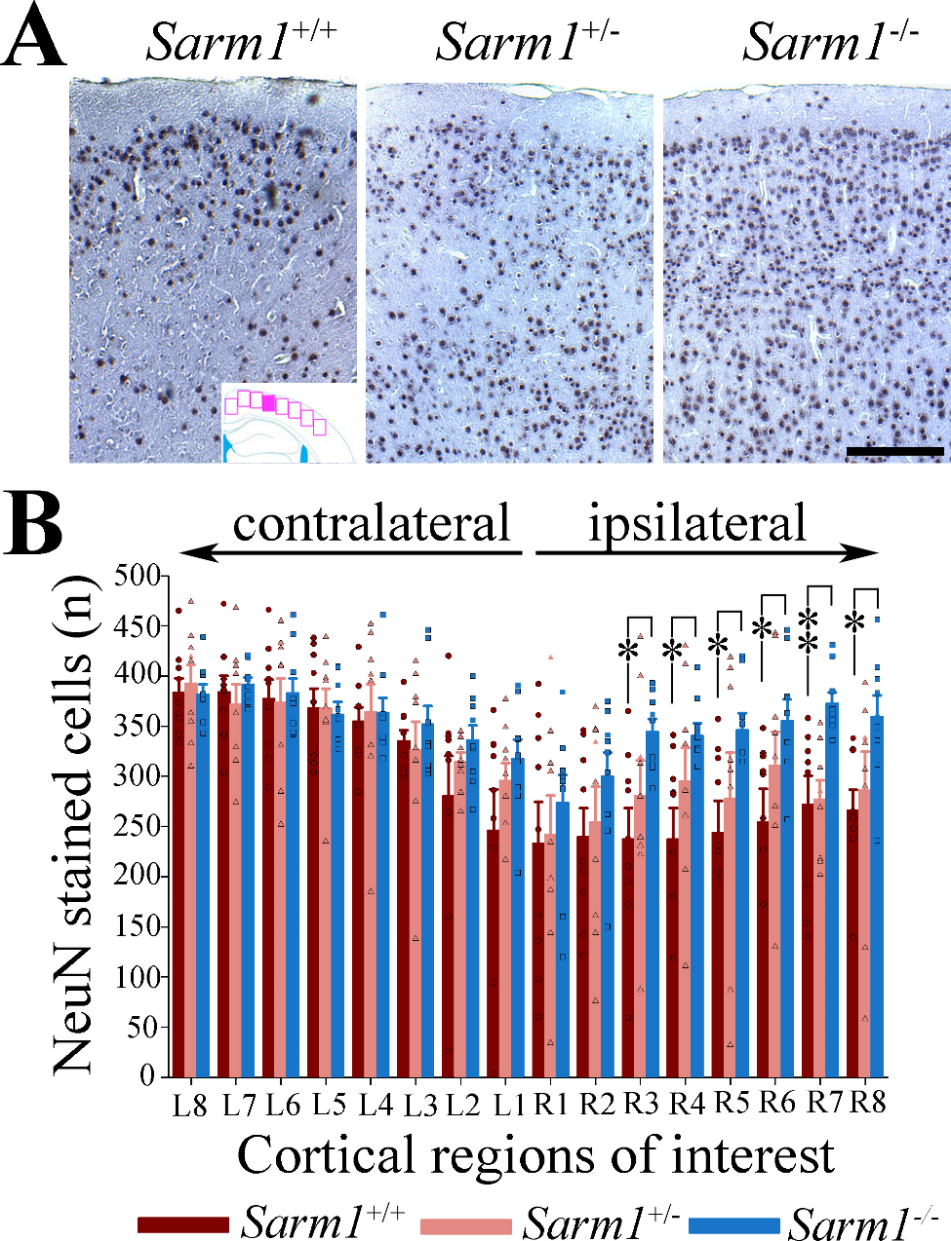
rTBI causes extensive neuronal loss at 1 month after injury that is attenuated by genetic ablation but not haploinsufficiency of *Sarm1*. (**A**) Loss of NeuN stained neurons in the cerebral cortex of *Sarm1*^*+/+*^ and *Sarm1*^*+/−*^ mice after rTBI (images were taken from an area approximately corresponding to the filled square in the inset; rectangles indicate the approximate region of interest (ROI) used for quantitative analyses shown in panel [B]). (**B**) Although *Sarm1*^*+/−*^ mice had numerically more neurons in the impacted hemisphere than *Sarm1*^*+/+*^ mice, this did not reach statistical significance. In contrast, genetic ablation of *Sarm1* significantly suppressed neuronal loss (*p* = 0.028 for group effect, *p <* 0.001 for ROI, *p* = 0.010 for group x ROI interaction). Data in the bar graph are shown as mean ± sem. n = 9 per group. *p < 0.05. **p < 0.01. Scale bar = 50 μm

Consistent with our model characteristics [33], rTBI resulted in significant neuronal loss within the ipsilateral cerebral cortex of wild type mice (Fig. 2A). Extending on prior observations in mild murine TBI models [7, 26, 51, 52], we now show that genetic ablation of *Sarm1* also substantially attenuated neuronal loss in our moderate-to-severe rTBI model when compared to *Sarm1*^+/+^ animals (Fig. 2A-B). Although *Sarm1*^+/−^ mice had numerically more NeuN positive profiles than *Sarm1*^+/+^ mice, this difference was not statistically significant (*p* = 0.307).

**Fig. 3:**
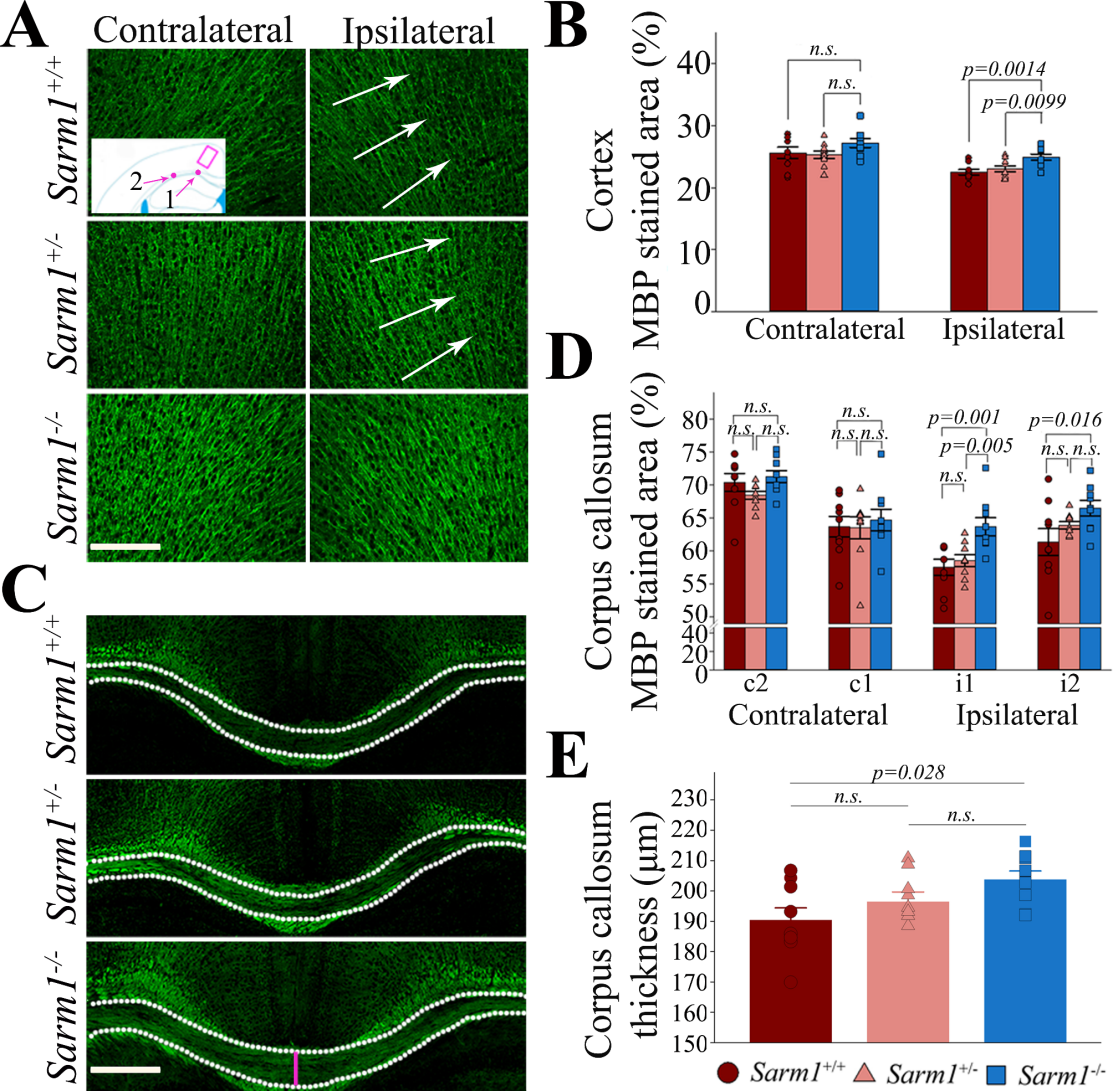
Genetic ablation of *Sarm1* attenuates loss of myelin staining in the cerebral cortex and corpus callosum as well as mitigates corpus callosum atrophy at 1 month after rTBI. Representative myelin staining (MBP) from the (**A**) cerebral cortex and the (**D**) corpus callosum at 1 month after rTBI with corresponding quantified signal from (**B**) one region of interest (ROI) in the cerebral cortex (square in inset) and (**C**) two ROIs in the corpus callosum (dots in inset). *Sarm1*^*−/−*^ mice had significantly greater MBP staining signal within the ipsilateral cortex when compared to *Sarm1*^*+/+*^ and *Sarm1*^*+/−*^ (*p* < 0.001 for side effects, *p* = 0.032 for group effects, *p* = 0.513 for group x side interaction, arrows indicate region with attenuation of the MBP staining signal indicating axonal rarefaction). Similarly, genetic ablation of *Sarm1* significantly attenuated myelin loss within the ipsilateral corpus callosum beneath the impact (ROI i1) and lateral to the impact (i2) (*p* = 0.023 for group effects, *p* < 0.001 for ROI effects, *p* = 0.079 for group x ROI interaction). There was no difference in myelin staining between groups in the corresponding contralateral ROIs (c1 and c2). (**E**) *Sarm1*^*−/−*^ mice had less corpus callosum atrophy (measured in the mid-sagittal plane, pink line) when compared to *Sarm1*^*+/+*^ mice (One-way ANOVA with *post hoc* Holm-Šidák test). All data are mean ± sem; n = 9 per group. Scale bars = 200 μm

We next sought to determine whether *Sarm1*^*−/−*^ and *Sarm1*^*+/−*^ mice have less axonal degeneration than wild type mice. We first quantified the MBP signal in the cerebral cortex underlying the impact area as well as in the corresponding contralateral cortex. We found that *Sarm1*^*−/−*^, but not *Sarm1*^*+/−*^ mice, had significantly preserved cortical MBP staining as compared to wild type mice (Fig. 3A-B). Likewise, when we quantified the MBP signal within the corpus callosum, we found that *Sarm1*^*−/−*^, but not *Sarm1*^*+/−*^, mice exhibited significantly preserved MBP staining within the ipsilateral corpus callosum (Fig. 3C-D). Finally, when we assessed the corpus callosum width in the mid-sagittal plane as a measure of atrophy on MBP-stained section, we found that *Sarm1*^*−/−*^ mice had significantly less corpus callosum atrophy when compared to *Sarm1*^+/+^ mice (*p* = 0.028). In contrast, no protective effect was observed in *Sarm1* haploinsufficient mice (*p* = 0.21, Fig. 3E). Results were similar when we used LFB-stained sections (Supplementary Fig. 1). Together, these results indicate that genetic ablation preserves cerebral axonal integrity after rTBI.

### Genetic ablation of ***Sarm1*** attenuates ipsilateral microgliosis and may reduce glial scarring

**Fig. 4:**
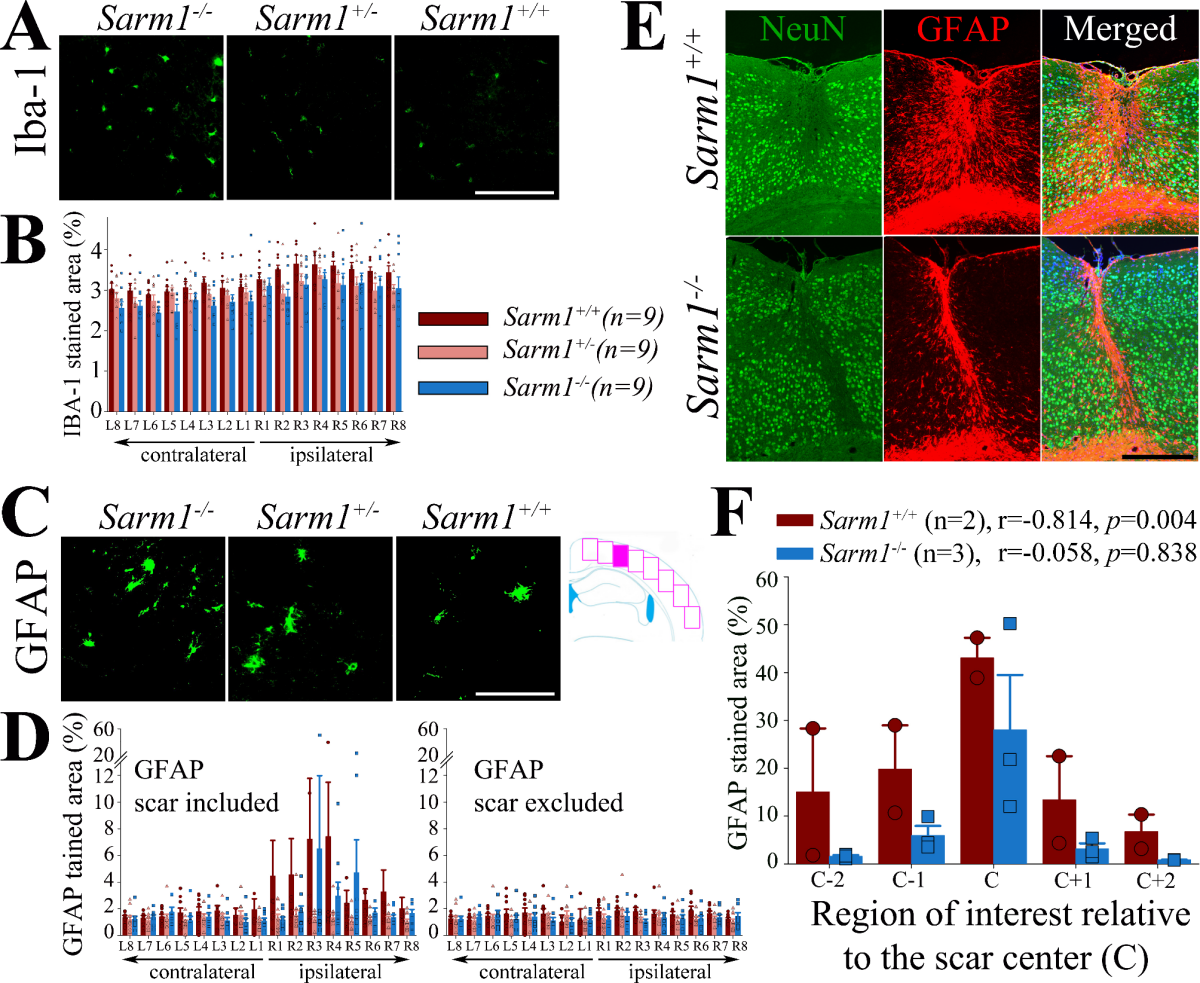
Genetic ablation of *Sarm1* attenuates cortical microgliosis and astroglial scar formation. (**A-B**) At 1 month after rTBI, there were significantly more Iba-1-stained microglia in the injured versus non-injured cortex in all groups (*p* = 0.045 for group effects, *p* = 0.001 for ROI effects, *p* = 0.299 for group x ROI interaction), whereby this effect was attenuated in both *Sarm1*^*+/−*^ (*p* = 0.028) and *Sarm1*^*−/−*^ (*p* = 0.034) mice. (**C-D**) We observed focal astrogliosis in the injured cortex of *Sarm1*^*+/+*^ and *Sarm1*^*−/−*^, but not *Sarm1*^*+/−*^, mice. After exclusion of the glial scar from analysis, we found no difference in the GFAP-staining signal between hemispheres and groups (*p* = 0.145 for group effects, *p* = 0.071 for ROI effects, *p* = 0.605 for group x ROI interaction). (**E**) Representative micrographs showing the glial scar of *Sarm1*^*+/+*^ and *Sarm1*^*−/−*^ mice with (**F**) corresponding quantification of the GFAP signal and correlation between GFAP and NeuN staining signal. Data in bar graphs are mean ± sem. n = 9 per group. Scale bars correspond to 100 μm in (A and C) and 500 μm in (E)

We found that rTBI caused ipsilateral microgliosis in the cerebral cortex of all groups, which was significantly attenuated in both *Sarm1* knockout and *Sarm1* haploinsufficient mice (Fig. 4A-B).

We also noted increased astroglial activation in the ipsilateral cerebral cortex of *Sarm1*^*+/+*^ and *Sarm1*^*−/−*^ mice as assessed by GFAP immunostaining in the ipsilateral cortex while no increase was observed in *Sarm1*^*+/−*^ mice (Fig. 4D). Upon further analysis, we found that this increase was driven by focal astroglial scar formation in a small subset of *Sarm1*^*+/+*^ (n = 2) and *Sarm1*^*−/−*^ (n = 3) mice rather than a global increase in the staining signal (Fig. 4C-D). Of note, no *Sarm1*^+/−^ mouse had an astroglial scar explaining the apparent lack of an increase in the GFAP signal as compared to the other groups. Indeed, when the cortical ROIs covering the cortical scar were omitted from the analyses, there was no significant difference in the degree of astroglial staining signal between *Sarm1*^*+/+*^, *Sarm1*^*+/−*^, and *Sarm1*^*−/−*^ groups (Fig. 4D).

Interestingly, when we specifically compared the GFAP signal taken from the ROIs centred around the astroglial scar, we found that the extent of astrogliosis was significantly smaller in *Sarm1*^−/−^ mice (Fig. 4E-F). Moreover, while there was an inverse correlation between the number of NeuN positive cells and GFAP within the area showing astroglial scar formation in between *Sarm1*^*+/+*^ mice (*r*=-0.814, *p* = 0.004) there was no correlation between the NeuN and GFAP signal in *Sarm1*^*−/−*^ animals (*r*=-0.058, *p* = 0.838). These data suggest that genetic ablation of *Sarm1* attenuates neuroinflammation and may reduce focal astroglial scar formation and associated neuronal loss after TBI.

### Both ***Sarm1*** knockout and ***Sarm1*** haploinsufficiency reduce cortical TDP-43 pathology after rTBI

**Fig. 5:**
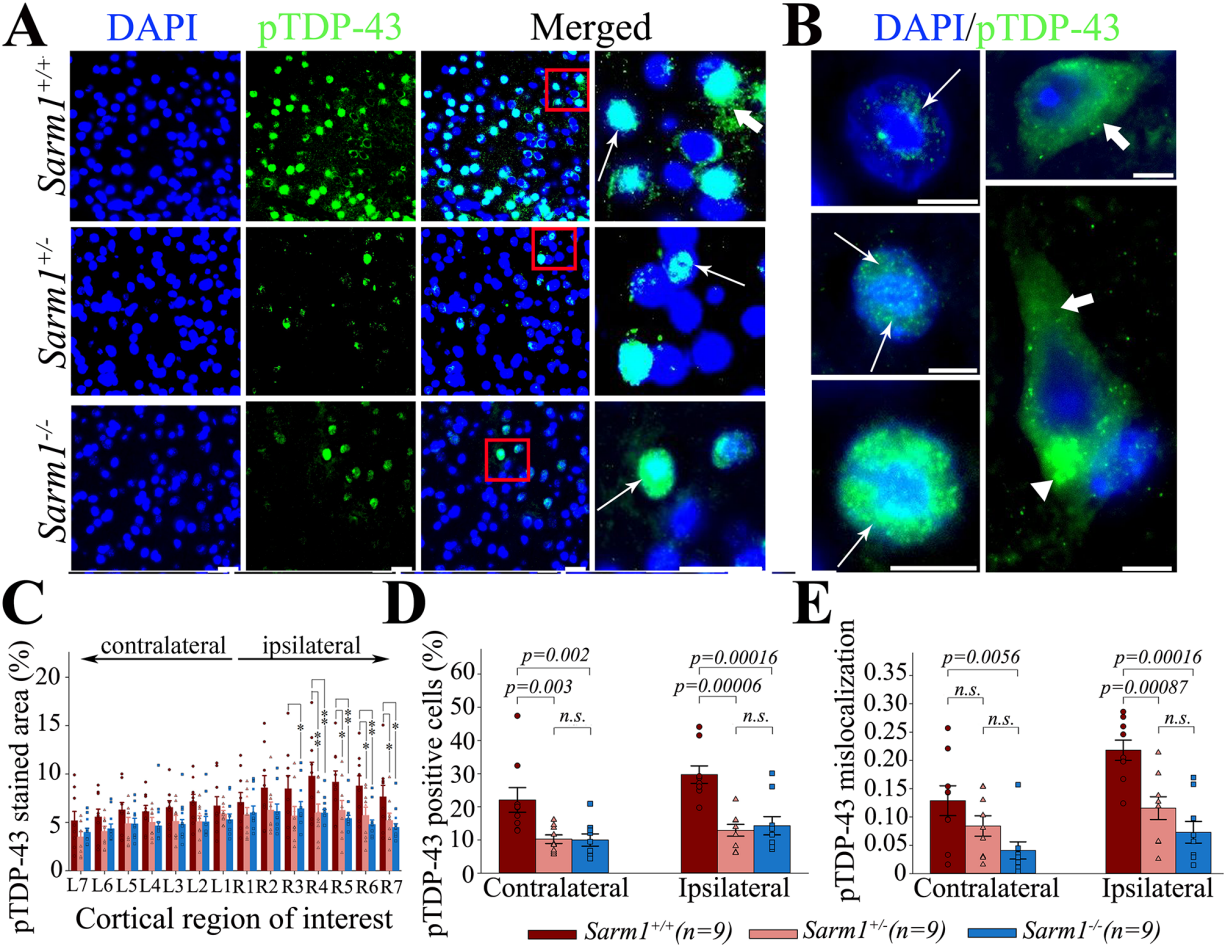
*Sarm1* knockout and haploinsufficiency suppress TDP-43 pathology after rTBI. Representative micrographs showing (**A**) cortical pTDP-43 expression in *Sarm1*^+/+^, *Sarm1*^*−/−*^, and *Sarm1*^*+/−*^ mice as well as (**B**) examples of neurons with different degrees of nuclear expression (long arrows), cytoplasmatic mislocalization (short arrows), and cytoplasmatic accumulation (arrowhead) of pTDP-43 in *Sarm1*^+/+^ mice. Compared to wild type *Sarm1*^+/+^ mice, *Sarm1*^*−/−*^ and *Sarm1*^*+/−*^ mice had significantly (**C**) attenuated pTDP-43 staining and (**D**) fewer pTDP-43 positive cells in the injured cerebral cortex at 1 month after rTBI. (**E**) Both *Sarm1*^*−/−*^ and *Sarm1*^*+/−*^ mice had significantly suppressed cytoplasmatic mislocalization of pTDP-43 in the injured cortex at 1 month. While there was also a reduction in pTDP-43 mislocalization within the non-impacted hemisphere, this effect was only significant in *Sarm1*^*−/−*^ mice. Data are mean ± SEM; n = 9 per group. *p < 0.05, **p < 0.01. Scale bars correspond to 25 μm in (A) and 50 μm in (B)

Persistent or irreversible cytoplasmic accumulation of TDP-43 is a major pathological event in several TBI-associated degenerative diseases [49, 54]. TDP-43 interacts with many different RNA, DNA, and protein targets [63]. While TDP-43 is mainly localized in the nucleus with only a small proportion located in the cytoplasm under physiological conditions, it is mislocalized to the cytoplasm after neuronal injury [63, 77]. Here, we found a significant increase in the pTDP-43 staining signal in the cerebral cortex of wild type mice at 1 month after rTBI, which was significantly suppressed in *Sarm1*^*+/−*^ and *Sarm1*^*−/−*^ mice (*p* = 0.024 for group effect, *p <* 0.001 for ROI, *p* = 0.747 for group x ROI interaction, Fig. 5A-C). Similarly, the number of pTDP-43 expressing cells was significantly greater in *Sarm1*^*+/+*^ mice as compared to *Sarm1*^*+/−*^ and *Sarm1*^*−/−*^ animals (*p < 0.001* for group effect, *p <* 0.001 for side effect, *p* = 0.206 for group x side interaction, Fig. 5D). On a cellular level, we found that *Sarm1*^*+/+*^ mice exhibited a prominent loss of nuclear pTDP-43 with cytoplasmic mislocalization and accumulation in both the ipsilateral as well as contralateral cerebral cortex at 1 month after rTBI that was significantly attenuated in *Sarm1*^*+/−*^ and *Sarm1*^*−/−*^ animals (*p < 0.001* for group effect, *p <* 0.001 for side effect, *p* = 0.154 for group x side interaction, Fig. 5E).

### Reduced expression of pTau in ***Sarm1***^-/-^ and ***Sarm1***^+/-^ mice after rTBI

**Fig. 6:**
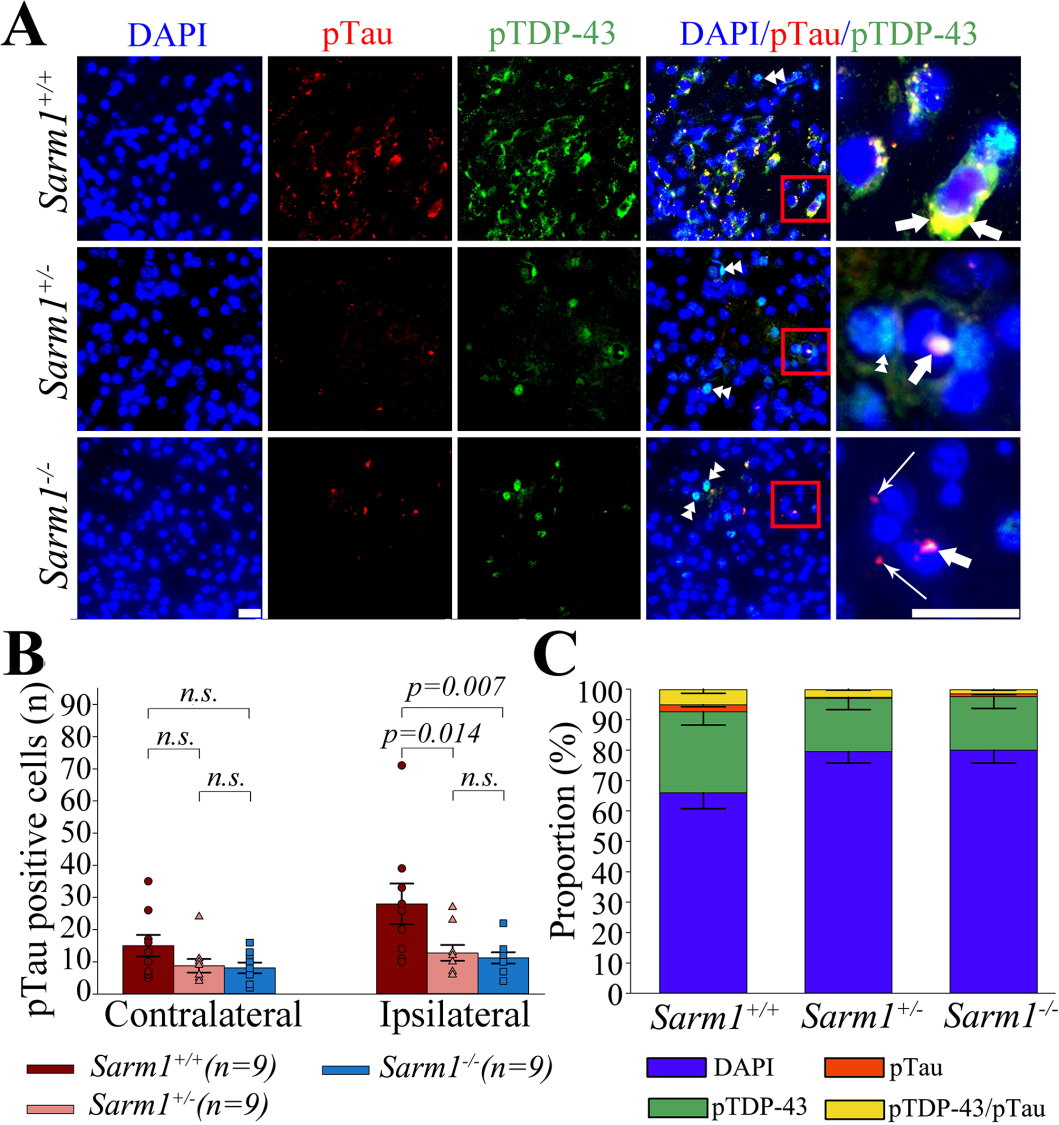
*Sarm1* knockout and haploinsufficiency suppress pTau expression after rTBI. (**A**) Representative micrographs showing the distribution of pTau versus pTDP-43 positive cells in the cerebral cortex at 1 month after rTBI. (**B**) Compared to wild type *Sarm1*^+/+^ mice, *Sarm1*^*−/−*^ and *Sarm1*^*+/−*^ mice had significantly fewer pTau positive cells in the injured cerebral cortex (long arrows indicate pTau stained cells, double arrowhead indicate pTDP-43 stained cells, and short arrow indicates pTDP-43/pTau double stained cells). (**C**) Shift towards fewer pTDP-43, pTau, and pTDP-43/pTau-double stained in *Sarm1*^*−/−*^ and *Sarm1*^*+/−*^ mice. While the proportion of pTDP-43 positive cells was similar for *Sarm1*^*−/−*^ (17.0%) and *Sarm1*^*+/−*^ (16.8%) groups, there were significantly fewer pTau and pTDP-43/pTau-double stained cells in *Sarm1*^*−/−*^ (2.3%) versus *Sarm1*^*+/−*^ (2.9%) (*p* < 0.05, χ^2^-test with *post-hoc* Bonferroni adjustment). Data are mean ± SEM; n = 9 per group. Scale bars = 50 μm

In addition to TDP-43 pathology, TBI initiates several non-mutually exclusive mechanisms that can lead to phosphorylation and accumulation of the protein Tau [28, 60, 81]. It has been recognized that pathological accumulation of pTDP-43 and pTau can be present in the brain of a single individual but little is known about the specific association of pTau versus pTDP-43 pathology in affected cells [33, 54, 59] and it is presently unknown whether targeting *Sarm1* affects post-traumatic pTau accumulation. Consistent with our prior observations [33], we found that wild-type mice had significantly more pTau positive cells in injured versus non-injured cortex at 1 month after rTBI (*p* < 0.001; Fig. 6A-B). Strikingly, the number of pTau positive cells in the injured cortex was significantly lower in both *Sarm1*^*+/−*^ and *Sarm1*^*−/−*^ mice when compared to wild type animals (*p* = 0.021 for group effect, *p <* 0.001 for side, *p* = 0.029 for group x side interaction, Fig. 6A-B). Consistent with the quantitative analyses, there was an overall shift towards fewer pTDP-43, pTau, and pTDP-43/pTau-double stained cells in both *Sarm1*^*−/−*^ and *Sarm1*^*+/−*^ mice (Fig. 6C). Nevertheless, only a small minority of cells expressed both pTDP-43 and pTau (5.1 ± 1.3 in *Sarm1*^*+/+*^, 2.6 ± 0.3 in *Sarm1*^*+/−*^, and 1.5 ± 0.3 in *Sarm1*^*−/−*^; Fig. 6C). Lastly, while the proportion of pTDP-43 positive cells was similar for *Sarm1*^*−/−*^ (17.0%) and *Sarm1*^*+/−*^ (16.8%) groups, there were significantly fewer pTau and pTDP-43/pTau-double stained cells in *Sarm1*^*−/−*^ (2.3%) versus *Sarm1*^*+/−*^ (2.9%) (*p* < 0.05, χ^2^ test with *post-hoc* Bonferroni adjustment) (Fig. 6C).

### ***Sarm1***^-/-^ mice had lower plasma IL-6 and CXCL1 levels at 1 month after rTBI as compared to ***Sarm1***^+/+^ mice

Given the observed activation of microglia, which release several proinflammatory cytokines in response to TBI [27, 40, 72, 78], we sought to determine whether genetic ablation of *Sarm1* affected plasma cytokine levels at 1 month after rTBI. We found that only IL-12p70 and IL-23 were elevated above the expected endogenous levels and without significant between-group differences (*p* > 0.05, each). However, though within the reference range, *Sarm1*^−/−^ mice had significantly lower levels of IL-6 (*p* = 0.038) and CXCL1 (*p* = 0.002) as compared to *Sarm1*^+/+^ mice (Supplemental Fig. 2).

## Discussion

Several studies established the role of the protein SARM1 in promoting axonal degeneration after various types of axonal injury including after TBI [7, 26, 51, 52]. Indeed, axonal degeneration is an early pathology in TBI that drives many of the observed functional deficits. It is presently thought that axonal injury may also play a role in the initiation of several processes such as the pathological accumulation TDP-43 that may promote chronic neurodegeneration [17, 24, 38, 57]. Using an established mouse rTBI model that replicates many aspects of human disease [33], we now provide proof-of-concept that blocking the SARM1-mediated prodegenerative pathway effectively attenuates the expression and mislocalization of pTDP-43 as well as accumulation of pTau in the cerebral cortex after injury.

A direct relation between TDP-43 and axon biology has recently been defined through the observation that TDP-43 is essential for the normal splicing and function of the axon maintenance factor **STMN2** [3, 37, 55]. However, as indicated above, recent data indicates that STMN2 does not regulate the activity of SARM1 [80]. Moreover, the mechanism by which TDP-43 prevents mis-splicing of STMN2 is not conserved in mice [55]. Indeed, it has been shown that *Sarm1* knockout did not rescue the motor phenotype associated with loss of the axon maintenance factor STMN*2* indicating that STMN2 does not regulate the activity of SARM1 in mice. Together these data suggest that TDP-43, SARM1, and neuronal degeneration after murine rTBI are interlinked by different, parallel pathways. For example, SARM1 activity may be in part regulated through c-Jun N-terminal Kinase (**JNK**). JNK is activated after axonal injury as well as by TDP-43 and, once activated, can bind and phosphorylate STMN2, promoting its degradation as well as enhance **NAD +** cleavage activity of SARM1 [58, 70, 75]. Conversely, it has been suggested that depletion of the natural calpain inhibitor calpastatin occurs downstream of the SARM1-dependent degeneration signal [88]; depletion of calpastatin accelerates TDP-43 cleavage and mislocalization to the cytoplasm [87]. Together, this provides a potential explanation for the improved TDP-43 pathology in SARM1 deficient mice, raising the possibility that blocking the *Sarm1*-mediated pathway could attenuate chronic TDP-43-mediated neurodegeneration after rTBI.

Substantial epidemiological data indicates that TBI is an important risk factor for several progressive neurodegenerative disorders including Alzheimer’s disease, frontotemporal dementia (FTD) and amyotrophic lateral sclerosis (ALS) [11, 13, 24, 36, 46, 47, 64, 83], particularly in individuals with a history of moderate-to-severe or repeated injuries [24, 48]. Evidence linking TBI to these conditions includes axonal degeneration as well as the pathological accumulation of proteins such as TDP-43 and Tau protein. Specifically, mislocalization and deposition of TDP-43 is a common neuropathological features in both TBI and ALS/FTD [44, 50, 82]. We recently demonstrated that rTBI can be an environmental risk factor that is sufficient to trigger ALS/FTD-associated neuropathology including widespread TDP-43 mislocalization and behavioral deficits in a transgenic mouse model of C9orf72 ALS/FTD that are not observed in non-transgenic and sham-operated control mice [35]. Accordingly, our findings may be highly relevant for developing anti-SARM1 therapies to mitigate the devastating consequences of these neurodegenerative disease. This notion is further supported by recent findings that SARM1 plays a role in ALS and FTD. While genetic ablation of *Sarm1* was not neuroprotective in the SOD1^G93A^ ALS mouse model [62], which typically lacks prominent TDP-43 pathology, this intervention did reduce neuron and axon loss in a transgenic TDP-43^Q331K^ mouse model of ALS/FTD [85]. Moreover, in ALS patients harboring rare SARM1 variants lacking normal autoinhibition, aberrant activation of SARM1 lead to neuronal degeneration in response to mild stress [5, 21].

Besides pathological TDP-43 accumulation, a potential link between axon injury and Tau pathology after rTBI has gained increasing attention in the field [24, 65]. Under physiological conditions Tau protein is present in the cytoplasm of axons exerting important function in microtubule stabilization and axonal transport [28, 81]. Since many TBI-associated neurodegenerative disorders are characterized by the accumulation of pathologic Tau it has been hypothesized that axonal injury may trigger the formation of pTau and thus represent a possible early step in the cascade that ultimately leads to pathological pTau accumulation and neurodegeneration [11, 24, 60, 81]. If true, inhibiting axonal degeneration could represent a viable approach to prevent Tau-mediated neurodegenerative disease [65]. We now provide proof-of-concept that blocking the prodegenerative *Sarm1* pathway may indeed mitigate pTau accumulation. Nevertheless, it is important to recognize that although the presence of pTau is considered an early event, further study is required to determine whether blocking *Sarm1* may indeed interrupt the formation of small, soluble oligomeric tau species and their aggregation into larger insoluble filaments known as neurofibrillary tangles, which represent the pathological hallmark of tauopathies [28].

Importantly, we found that genetic removal of *Sarm1* significantly attenuated neuronal loss, axonal degeneration, and neurological deficit severity after *moderate-to-severe* rTBI. It has previously been shown that inactivation of *Sarm1* attenuates pathology after mild TBI but it was heretofore unknown whether targeting *Sarm1* could be beneficial for mitigating sequelae of severe TBI. The importance of this issue is highlighted by data from the prospective, multicenter observational TRACK-TBI (Transforming Research and Clinical Knowledge in TBI) study. This study showed that by one year after TBI approximately 50% of patients with severe and 40% of patients with moderate TBI experienced an unfavourable outcome (death or dependence on daily assistance) whereby the cumulative 1-year mortality reached 30.6% after severe and 13% after moderate TBI [53]. In this regard it is striking that *Sarm1* knockout mice had a 35% absolute risk reduction in death, showing for the first time that genetic ablation of *Sarm1* significantly improves survival after TBI. Although direct translation from our model to the clinic is not possible, it is noteworthy that in TRACK-TBI most deaths occurred within the first 2 weeks; similar to our model in which no mice died after 14 days. This raises the tantalizing possibility that *Sarm1* targeting therapies may be a viable approach to improve both disability and overall survival after TBI.

The NADase activity of SARM1 appears to correlate with the gene dosage. It has been found that the SARM1-dependent breakdown of NAD + to cyclic adenosine diphosphate ribose (cADPR) after neve injury is proportional to SARM1 gene dosage as *Sarm1* heterozygous mice showed an approximately 50% reduction in the level of cADPR compared to wild type animals [67]. This is an important observation as it is unlikely that pharmacological strategies targeting SARM1 will completely remove the protein and its activity and most prior studies did not observe a durable neuroprotective effect of *Sarm1* haploinsufficiency after neuronal injury [19, 23, 61]. Yet, recent important observations in the field indicated that both *Sarm1* haploinsufficiency and partial blockage of *Sarm1* attenuated axon degeneration in vitro as well as in vivo peripheral nerve injury models [19, 23]. Together, these observations suggested that the efficacy of incomplete *Sarm1* blockage to mitigate nerve injury may depend on the type and severity of insult and it remained to be shown whether partial *Sarm1* inactivation could mitigate the effects of central nervous system injury. A second important result of our study was that removing one *Sarm1* allele transiently suppressed neurological deficits and only non-significantly attenuated neuronal and axonal loss without improving mortality. This suggests that reducing *Sarm1* function by 50% may delay, but ultimately not prevent, early neuronal injury in moderate-to-severe rTBI. Accordingly, any *Sarm1*-based therapy will likely have to reduce function by more than 50% to achieve a durable effect on early neuropathology after acute brain injury. There has been highly encouraging progress made in the development of anti-SARM1 therapeutics, some of which have been found to achieve sufficient suppression of SARM1 activity to prevent axon degeneration after injury in vitro [6, 8, 30], as well as in mouse models of peripheral nerve injury [6, 8]. Moreover, in vitro data indicates that axon degeneration can be significantly attenuated even when treatment is delayed by several hours [30]. After axonal injury, a substantial proportion of affected axons exist in a metastable state for some time and have the potential to either recover or progress into irreversible degeneration [30, 86]. Administration of a SARM1 inhibitor during the metastable period has been shown to achieve axon recovery indicating a window of opportunity to initiate therapy after the original injury [30]. Lastly, in the context of TBI genetic ablation of *Sarm1* has been found to not only preserve axon integrity acutely and in the immediate vicinity of the impact but also to attenuate axon degeneration occurring remotely from the injury site that persisted to the subacute phase [2]. Together, these and our observations indicate that targeting SARM1 may offer an effective neuroprotective strategy against acute and subacute axonal degeneration after TBI.

TBI is associated with a dynamic inflammatory response that may remain active long after the original injury [27, 40, 72, 78]. We made the striking observation that both *Sarm1* knockout and *Sarm1* haploinsufficiency significantly attenuated microgliosis as well as pTDP-43 and pTau accumulation in the cerebral cortex after injury. Adverse interactions with non-neuronal cells such as microglia are presently understood to play in important role in chronic neurodegenerative processes after TBI and associated neurodegenerative diseases [4, 9, 69]. Limited data indicate that glial activation may precede TDP-43 mislocalization and accumulation [49] and disease-causing mutations in microglia promote TDP-43 aggregation and cell death, suggesting that TDP-43 proteinopathy and neurodegeneration are interlinked with chronic microglial activation [89]. In addition to its involvement in axon degeneration, SARM1 plays a key role in innate immunity and inhibition of SARM1 has been shown to attenuate microgliosis in multiple disease models [43, 76, 90] including TBI [2, 15, 52]. Importantly, genetic ablation of *Sarm1* prevented axonal degeneration in the spinal cord tracts and the accompanying neuroinflammatory response that extended into the subacute phase after TBI [2]. Moreover, compound heterozygous mice for the human NMNAT2^V98M^ and NMNAT2^R232Q^ mutations develop progressive motor dysfunction, peripheral axon loss, and macrophage infiltration [15]. In this model, genetic ablation of *Sarm1* prevented axon degeneration and reduced macrophage activation and macrophage depletion therapy blocked and reversed neuropathic phenotypes, identifying a SARM1-dependent neuroimmune mechanism as a key driver of disease pathogenesis [15]. Lastly, the injury cascade after TBI includes the release of various pro- and anti-inflammatory cytokines; *Sarm1* knockdown has been shown to alter cytokine levels both in the absence [42] and presence of an inflammatory response [29, 56, 76, 84]. We found that at 1 month after rTBI *Sarm1* knockout mice had significantly lower levels of IL-6 and CXCL1. After brain injury both IL-6 and CXCL1 levels typically peak at 1 day with rapid decline by days 2–3, and it has been proposed that their upregulation after injury may play an important role in recruitment of microglia as well as peripheral leukocytes to the brain after injury [16, 32, 45, 72]. Nevertheless, some studies indicated that expression of certain cytokines including IL-6 may remain elevated for several days if not weeks after the original injury [40, 78]. Together these observations raise the intriguing possibility that *Sarm1* may in part impact chronic neuroinflammation after TBI via IL-6 and CXCL1and suggest that anti-SARM1 therapies could potentially mitigate TBI-associated neurodegenerative processes by attenuating injury associated neuroinflammation. Further study is required to determine the interaction of microglial activation, cytokine expression, and TDP-43 pathology after TBI and whether this intersects on a SARM1-dependent pathway.

Finally, we made the intriguing observation that *Sarm1* knockout mice had fewer seizures and attenuated glial scar formation. Astrogliosis in response to focal brain damage is thought to be an important mechanism to protect uninjured brain by sealing injured areas. Nevertheless, this process may result in impaired local ion and transmitter homeostasis increasing the risk for seizures, particularly once a glial scar has formed [68]. Indeed, mild non-proliferative astrogliosis has been shown in mouse rTBI [33, 68] and greater astrogliosis increased the risk for post-rTBI seizures [68]. Interestingly, *Sarm1* is thought to act upstream of the apoptosis signal-regulating kinase 1 (ASK1)-p38 pathway [14, 31], which has been shown to promote astrocyte-mediated inflammatory responses [25], thus providing a possible pathomechanistic link between *Sarm1* activation, astrogliosis, and seizures in TBI.

## Conclusions

Here we demonstrate that blocking the prodegenerative *Sarm1*-pathway in moderate-to-severe rTBI attenuates the expression and mislocalization of pTDP-43, accumulation of pTau as well as mitigates neuronal and axonal injury and neuroinflammation, improves neurological deficit severity and survival. We show that genetic removal of only one *Sarm1* allele delays, but ultimately does not prevent, functional deficits, death, and neuronal and axonal degeneration. Therefore, pharmacological strategies targeting SARM1 to prevent the acute effects of rTBI likely need to reduce efficacy by more than 50%. Our data suggest that partial inactivation of *Sarm1* is sufficient to reduce the pathological accumulation of pTDP-43 and pTau. Further studies are required to determine whether this could attenuate chronic neurodegenerative and neuroinflammatory processes after acute brain injury.

### Electronic supplementary material

Below is the link to the electronic supplementary material.

**Supplementary Material 1: Fig. S1** Genetic ablation of Sarm1 attenuates corpus callosum atrophy at 1 month after rTBI. **Fig. S2** Plasma cytokine levels at 1 month after rTBI

## Data Availability

Not applicable.

## List of abbreviations

ALS: Amyotrophic lateral sclerosis
ANOVA: One-way analysis of variance
ARM: HEAT/Armadillo motif
ASK1: Apoptosis signal-regulating kinase 1
C9orf72: Chromosome 9 open reading frame 72
cADPR: Cyclic adenosine diphosphate ribose
CMOS: Complementary metal-oxide semiconductor
CXCL1: C-X-C motif chemokine ligand 1
EDTA: Ethylenediaminetetracetic acid
FDR: False discovery rate
FTD: Frontotemporal dementia
GFAP: Glial fibrillary acidic protein
Iba-1: Ionized calcium binding adaptor molecule 1
IL: Interleukin
JNK: C-Jun N-terminal Kinase
LFB: Luxol fast blue
MAPK: Mitogen-activated protein kinase
MBP: Myelin basic protein
NAD +: Nicotinamide adenine dinucleotide
NeuN: Neuronal nuclei
NMN: Nicotinamide mononucleotide
NMNAT2: Nicotinamide nucleotide adenylyltransferase 2
NSS: Neurological severity score
pTau: Phosphorylated Tau
pTDP-43: Phosphorylated transactive response DNA binding protein 43 kDa
ROI: Region of interest
RRID: Research resource identifiers
rTBI: Repetitive traumatic brain injury
SARM1: Sterile alpha and TIR motif containing 1
sCMOS: Scientific complementary metal–oxide–semiconductor
SOD1^G93A^: G93A-superoxide dismutase-1
STMN2: Stathmin2
TBI: Traumatic brain injury
TDP-43: Transactive response DNA binding protein 43 kDa
TIR: Toll-interleukin-1 receptor
TRACK-TBI: Transforming Research and Clinical Knowledge in TBI
