# Supplementary material for: Genetic ablation of Sarm1 attenuates expression and mislocalization of phosphorylated TDP-43 after mouse repetitive traumatic brain injury
Source: Acta Neuropathol Commun. 2023 Dec 20;11:206. doi: 10.1186/s40478-023-01709-4 (PMC10731794; doi:10.1186/s40478-023-01709-4)
Supplement: Supplementary file 1 — Supplementary Material 1: Fig. S1 Genetic ablation of Sarm1 attenuates corpus callosum atrophy at 1 month after rTBI. Fig. S2 Plasma cytokine levels at 1 month after rTBI [file 40478_2023_1709_MOESM1_ESM.docx]

**Supplementary Information**

**Genetic ablation of *Sarm1* attenuates expression and mislocalization of phosphorylated TDP-43 after mouse repetitive traumatic brain injury**

**Running Title: *Sarm1* deficiency mitigates TDP-43 pathology in TBI**

**Authors:** Elif O. Dogan^1^, James Bouley^1^, Jianjun Zhong^1,2^, Ashley L. Harkins^1,3,4^, Allison M. Keeler^4,5,6^ Daryl A. Bosco^1^, Robert H. Brown Jr.^1^, Nils Henninger^1,7*^

**Affiliations:**

^1^Department of Neurology, University of Massachusetts Chan Medical School, Worcester, MA 01605, USA

^2^Department of Neurosurgery, The First Affiliated Hospital of Chongqing Medical University, Chongqing, China

^3^Graduate Program in Neuroscience, Morningside Graduate School of Biomedical Sciences, University of Massachusetts Chan Medical School, Worcester, MA 01605, USA

^4^Horae Gene Therapy Center, University of Massachusetts Medical School, Worcester, MA 01605, USA

^5^Department of Pediatrics, University of Massachusetts Medical School, Worcester, MA 01605, USA

^6^NeuroNexus Institute, University of Massachusetts Medical School, Worcester, MA 01605, USA^7^Department of Psychiatry, University of Massachusetts Medical School, Worcester, MA 01605, USA

***Correspondence to:** Nils Henninger, MD, PhD, Dr med

Departments of Neurology and Psychiatry
University of Massachusetts Chan Medical School
55 Lake Ave, North
Worcester, MA 01655
Tel: (774) 455-3760
Fax: (508) 856-2811
E-mail: nils.henninger@umassmed.edu

## *Supplemental Figure 1*


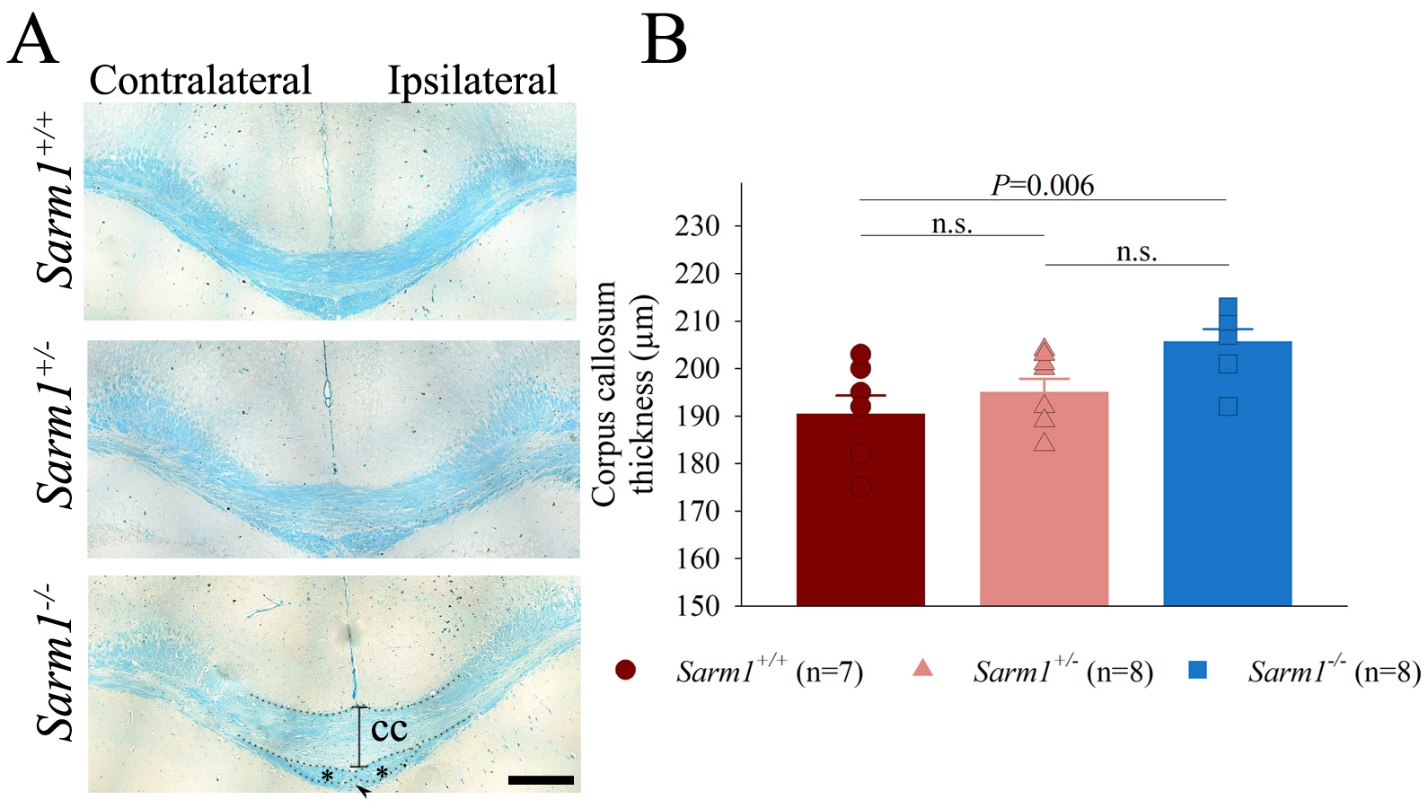


**Fig. S1 Genetic ablation of Sarm1 attenuates corpus callosum atrophy at 1 month after rTBI. (A)** Representative myelin staining Luxol fast blue from the corpus callosum (cc) at 1 month after rTBI. Corpus callosum atrophy was measured in the mid-sagittal plane (marker between dashed lines) above the dorsal fornix (asterisks) and dorsal hippocampal commissure (arrowhead). **(B)** *Sarm1^-/-^* mice had less corpus callosum atrophy when compared to *Sarm1^+/+^* mice (One-way ANOVA with *post hoc* Bonferroni adjustment). All data are mean±sem; Scale bars = 250 µm. Note that for presentation purposes, image contrast and brightness of the histological figures was enhanced by using Photoshop's nonlinear curve function across the entire image.

# *Supplemental Figure 2*


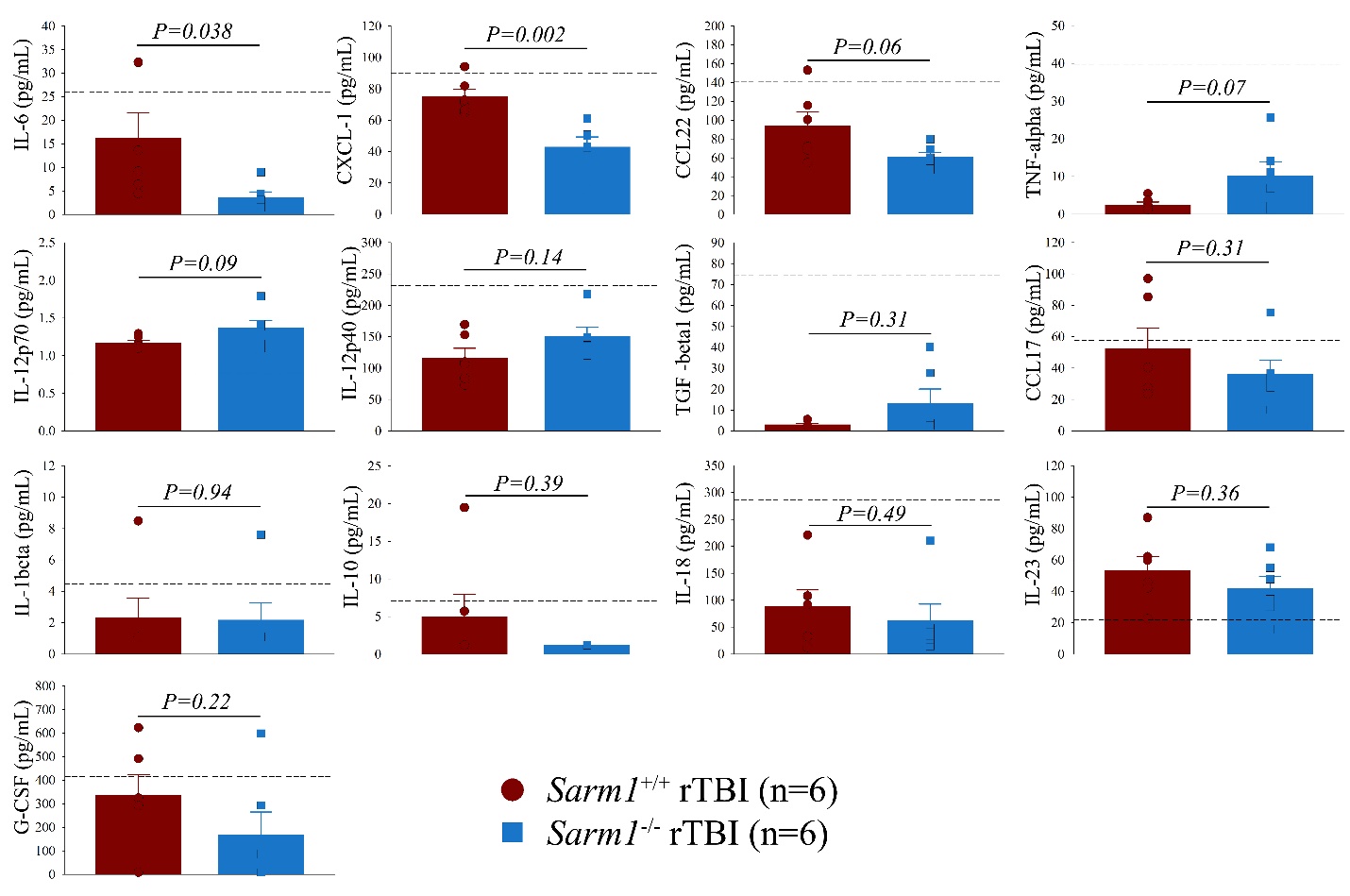


**Fig. S2 Plasma cytokine levels at 1 month after rTBI**. Level of detection is defined as follows for each analyte (CXCL1: 1.22, TGF-B1: 2.69, IL-18: 12.2, IL-23: 6.71, CCL22: 1.22, IL-10: 1.22, IL-12p70: 1.10, IL-6: 1.46, TNF-a: 1.22, G-CSF: 8.54, CCL17: 1.22, IL-12p40: 1.22, IL-1B: 1.10). Dashed lines indicate upper threshold for expected endogenous levels for C57Bl/6 mice. All data are mean±sem. All comparisons were made by t-Test.
